# Wastage of Blood Units at Tertiary Care Hospitals of Lahore

**DOI:** 10.7759/cureus.8040

**Published:** 2020-05-09

**Authors:** Mirza Iqra Baig, Aymen Javed, Muhammad Asif Naveed, Kashifa Nawab, Syed Anwaar Alam, Muhammad Asif

**Affiliations:** 1 Pathology, King Edward Medical University Lahore, Lahore, PAK; 2 Gynaecology and Obstetrics, Services Hospital Lahore, Lahore, PAK; 3 Pathology, Jinnah Hospital/Allama Iqbal Medical College (AIMC), Lahore, PAK; 4 Pathology, Alkhidmat Blood Bank, Surrayya Azeem Hospital, Lahore, PAK; 5 Pathology, Alkhidmat Blood Bank, Lahore, PAK

**Keywords:** cross match to transfusion ratio, maximum surgical blood ordering schedule, transfusion probability, transfusion index

## Abstract

Introduction: Blood and its products are the most valuable resource in every healthcare institution. Judicial use of these limited resources is necessary and important to maintain sufficient supply. Blood products and its transfusion system are being evaluated by the use of several markers. The use of crossmatch to transfusion ratio (C/T) was first proposed by Boral Henry in 1975. Nowadays, the C/T ratio is being used by researchers to assess transfusion practices. Ideally, (C/T) ratio has to be 1.0 but it has been suggested that a ratio of 2.5 or less would indicate effective blood use.

Objective: To assess crossmatch to transfusion ratio (C/T ratio), transfusion probability (%T), and transfusion index (TI) in patients of medical and surgical wards. To assess the wastage of blood components/whole blood units.

Methods: A cross-sectional study was conducted at Mayo Hospital/King Edward Medical University, Lahore, Services Hospital Lahore, and Alkhidmat Blood Bank Surrayya Azeem Hospital between January 2019 and June 2019. Nonprobability convenient sampling was performed and all collected data were entered and analyzed by using statistical package for social sciences (SPSS version 20).

Results: The total patients who were ordered to arrange blood were 1322 for which overall 2715 crossmatches had been done. Among these crossmatches, a total of 1536 of the donors were bled for from which only 815 units had been transfused to the patients. Overall C/T ratio was found to be 3.33 and the overall wastage was 38.9%.

Conclusions: Blood transfusion is considered useful in the management of surgical as well as medical patients. Too many units are ordered out of which the majority are wasted due to nonutilization. There is a need for standard operating procedures for transfusion of blood. There shall be regular audits to improve the blood collection, treatment, delivery, and usage practices of this scarce resource.

## Introduction

Blood and blood components are vital resources for any healthcare institution. To cope with ever-increasing demand and wastage of blood and blood products is a global challenge [1]. An unnecessary request for whole blood or red cell concentrate can lead to the consumption of reagents, time wastage that would otherwise be used for necessary requests. Such requests also place a financial burden on patients, as preoperative blood collection can increase material and human resources needs at medical institutions and thus increase medical expenses [2].

Several indicators can be used to evaluate the effective use of blood and blood components. The use of the crossmatch to transfusion (C/T) ratio, first proposed by Boral Henry in 1975, is now a widely accepted marker [3]. Ideally, the C/T ratio should be 1.0, but studies have indicated that any ratio of 2.5 or less indicates effective blood use [4]. The transfusion probability for any given treatment is expressed as a percentage (T%). Mead et al. first suggested transfusion probability in 1980 [5]. The transfusion index (TI) refers to the average number of units used per patient, which indicates the suitability of crossmatching the corresponding number of units. Values above 0.5 indicate an effective use of blood [6]

This study was designed to analyze the C/T ratio, transfusion probability (%T), and transfusion index (TI) at the blood bank of the three tertiary care hospitals of Lahore.

## Materials and methods

Study design and population

The cross-sectional study was carried at the blood bank of Mayo Hospital, blood bank of Services Hospital, and Alkhidmat Blood Bank Lahore between January 2019 and June 2019. Blood component transfusion requests for 1322 patients/recipients were included in this study by a nonprobability convenient sampling technique. Healthy donors meeting the WHO criteria for blood donation are included in this study. Any blood unit requested for chronic transfusion-dependent anemias of children like thalassemia and inherited aplastic anemia, as assessed using patient medical history, was excluded from the study.

Data collection procedure

The donors were enrolled using a proforma, which includes donor name, age, gender, religion, marital status, status of blood donation, and type of blood donation. Screening for infectious diseases was performed if negative, then blood was collected, crossmatched, and stored until issuance. The dispatched blood bags were tracked in wards to find out whether they were transfused or not. The number of expired blood bags was noted, and a comparison of the number of crossmatched to transfused blood bags was made. The date of withdrawal and the date of expiry both were noted on the blood bags and in the blood bank’s register. Total crossmatched and transfused units were counted and the data were computed using SPSS, version 23.0. The C/T ratio, transfusion probability, and TI values were calculated as follows.

Crossmatch to transfusion ratio (C/T ratio) = number of units crossmatched/number of units transfused

Transfusion index (TI) = number of units transfused/number of patients crossmatched

Transfusion probability (%T) = number of patients transfused × 100/number of patients crossmatched

A ratio of 2.5 or less was taken as effective blood usage. A value of 0.5 or more was considered as an effective transfusion index and a value of ≥30% was taken as the normal value for %T.

## Results

Transfusions were requested for a total of 1,322 patients, for which 2,715 cross matches were carried out. Socioeconomic data are presented in Table 1, below. Of these crossmatches, 1,536 of the donors were bled for a blood transfusion but only 815 units were transfused to the patients. The overall C/T ratio was 3.33, the transfusion probability was 33%, and the transfusion index was 0.61. Overall, 46.9% of blood units were left unused and 38.9% of the units were wasted. Only 8% of the units were used for patients on a nondonor basis. Most of the donors were students or relatives of the patients. Majority of the donations came from repeat/regular donors, as shown in Table 1.

**Table 1:** Sociodemographic and other characteristics of donors.

S.no	Characteristics		Total	Percentage (%)
1	Gender	Male	993	65
		Female	543	35
2	Age	Less than 20	96	6
		20-30	632	41
		30-40	491	32
		Greater than 40	317	21
3	Education level	No formal education	319	21
		Primary	329	21
		Secondary	316	21
		Tertiary	572	37
4	Blood donation status	First time	626	41
		Repeat	910	59
5	Type of donation	VNRBD	369	24
		Replacement	255	17
		Directed	912	59

In total, 22% of all requests were generated by the gynecology and obstetrics department, for which 703 crossmatches were conducted. There were 161 transfused units, equating to a C/T ratio of 4.3 in this department.

The requests generated by the ED accounted for 15.2% of all requests and 409 crossmatches. Of these, 245 donors were bled for transfusion and only 156 units were transfused, leading to a C/T ratio of 2.62 for emergency surgeries. A total of 206 requests were generated by patients in the elective surgery department and 485 crossmatches were carried out for these patients while 225 donors were bled for these. Of these 485 crossmatched units, only 136 were transfused, resulting in a C/T ratio of 3.5. This indicates an over-ordering of blood and misuse of blood bank resources.

For the oncology department, 326 requests were crossmatched, of which 105 were transfused, leading to a C/T ratio of 3.1. In general medicine, blood component requests for made for 226 patients and 498 crossmatches were carried out. A total of 262 donors were bled, from whom only 143 units were transfused. The C/T ratio was calculated as 3.4. The C/T ratio was 2.57 for the orthopedic department, for which 294 crossmatches were conducted and only 114 were transfused. These results are explained in detail in Table 2.

**Table 2:** Transfusion probability, transfusion index, and crossmatch to transfusion ratio.

#	Departments	Total patient for whom requests were generated	Crossmatches performed	Donor bled	Transfused units	Number of patients transfused	Crossmatch to transfusion ratio	Transfusion index	Transfusion probability (%)
1	Oncology	198	326	201	105	55	3.10	0.5	28
2	Emergency surgery	202	409	245	156	109	2.62	0.78	53
3	General medicine	226	498	262	143	69	3.48	0.6	30
4	Orthopedics	198	294	141	114	58	2.57	0.57	29
5	Elective surgery	206	485	225	136	61	3.5	0.6	29.6
6	Gynecology and obstetrics	292	703	462	161	84	4.3	0.5	28
	Total	1322	2715	1536	815	436	3.33	0.61	33

## Discussion

The increase in demand for blood and its products has led to many recent studies on blood supply and blood transfusion practices. In this study, a total of 1,322 requests were generated for crossmatches. For these requests, 2,715 crossmatches were performed and only 815 units were transfused so 1900 extra crossmatches were performed. This indicates the misuse of blood bank resources and reagents and demonstrates the increased burden on the blood bank itself.

In our setting, surgeons most often demand fresh blood and do not use preserved/refrigerated or older blood units during surgical procedures. This study revealed that 70% of the cross-matched units were not transfused, a result which is comparable to a study conducted in Nigeria that found 69.7% of units left unused [7]. This finding was also comparable to studies conducted in India and Egypt, where the equivalent figures were 76.8% and 74.8% respectively [8-9]. This high degree of wastage can be attributed to clinicians’ demands for fresh blood; medical practitioners ask the blood bank to bleed directed donors for fresh blood and if that blood is not used for that patient, it usually ends up being disposed of.

While an ideal C/T ratio is 1.0, it has been suggested that any ratio below 2.5 would indicate effective blood use [4]. In our present study, the overall C/T ratio was 3.33, which is above normal and indicates an ineffective use of blood and its products at this hospital. This ratio differed from the study conducted at Aga Khan Hospital, Karachi, Pakistan, in which the overall C/T ratio was less than 2.5 for 23 different procedures. This is because Aga Khan University Hospital follows the maximum surgical blood order schedule (MSBOS) blood conservation guidelines [10]. And no MSBOS is in place at tertiary care centers included in this study. 

In the current study, the highest C/T ratio was in the gynecology and obstetrics department, at 4.3. This finding is comparable to a study conducted in Saudi Arabia that revealed a gynecology and obstetrics C/T ratio of 5.2. After gynecology, the elective surgery department wasted the most blood. The C/T ratio in this department was 3.5, which is comparable to the study conducted in Ethiopia, where the greatest blood waste was revealed in the elective surgery department [11]. The high ratio was down to an increased tendency for surgeons to order more blood for elective surgeries that were needed. This over-ordering of blood might be due to a physician’s overestimation of blood needs for specific surgical procedures that are more likely to incur more significant blood loss. Many blood units were wasted for this reason. This over-ordering of blood can be avoided by creating specific blood transfusion guidelines similar to those in MSBOS.

The C/T ratio in cases of emergency surgery was 2.62, indicating a much more effective use of blood. The present study also revealed C/T ratios in oncology and general medicine as 3.1 and 3.48, respectively. This finding is quite different from those obtained from the study conducted in Rawalpindi, which examined blood cross-match ordering schedules. This study showed C/T ratios in oncology and general medicine as 1.08 and 1.1, respectively [12]. These findings indicate a more effective use of blood in their sectors and a reduced level of blood waste as compared to ours and indicate that we need to look into blood transfusion policies in our medical institutions.

Our study revealed an overall transfusion probability of 33%, as compared to studies by Devi et al., Kaur et al., and one in the Jammu region, which revealed %T values of 97.2%, 79%, and 88.8%, respectively, showing an effective utilization of blood [13-15]. 

## Conclusions

Our results show that the majority of cross-matched units were not transfused and that at least 38.9% of units were wasted. Wasting blood components or whole blood is higher because clinicians usually ask for fresh blood so the majority of the refrigerated blood components are wasted. The overall C/T ratio was greater than normal, indicating an ineffective use of blood and its components. Unnecessary ordering of blood increases the burden on blood banks and results in higher waste of reagents. It also results in wasted time for blood bank personnel. The use of a blood conservation policy like MSBOS could reduce unnecessary crossmatches.
